# Editorial: Understanding the link between environmental pollutants, brain & behavior

**DOI:** 10.3389/fpsyt.2026.1832093

**Published:** 2026-03-24

**Authors:** Liana Fattore, Marc Landry

**Affiliations:** 1 Institute of Neuroscience, Cagliari, National Research Council (CNR), Cagliari, Italy; 2 University of Bordeaux, Centre National de la Recherche Scientifique (CNRS), Institut des Maladies Neurodégénératives (IMN), Unité Mixte de Recherche (UMR), Bordeaux, France

**Keywords:** behavior, brain health, microplastics, neuroinflammation, oxidative stress, particulate matter, pollution

Environmental pollution is well known to be associated with poorer physical health and to worsen organic diseases, including some types of cancer, while less attention has been given so far to the consequences of exposure to pollutants on mental health. Mental diseases have become a societal and economic burden, with growing evidence associating environmental pollution with an enhanced risk of developing neurological conditions, psychiatric disorders, and comorbidities. It is becoming increasingly evident that many pollutants can affect the human nervous system with remarkable consequences for the population’s mental health. However, the biological mechanistic pathways underlying the effects of pollutants on brain integrity and functions remain elusive. To date, no therapeutic treatment is designed to target pollution-based mental disorders and associated neurological conditions, which makes this area of research a vital public health priority and highlights the need to understand the role of pollutants in mental illness to devise therapeutic approaches.

This Research Topic collects 6 Original Articles that include both preclinical and clinical studies, 1 Community Case Study article, 2 Reviews and 1 Mini Review, all focused on the interaction between environmental pollution and mental health. Pollutants under investigation included particulate matter (i.e., a mixture of solid particles and liquid droplets suspended in the air, including dust, soot, smoke, and chemicals, that vary in size), microplastics (i.e., small plastic particles ranging from 1 µm to 5 mm in size), organophosphate pesticides, indoor pollutants (e.g., volatile organic compounds), ethylene oxide (i.e., a reactive epoxide derived from ethylene), heavy metals, asbestos, pollutant emissions from brick kilns but also light and noise pollution. Mental conditions taken into consideration in this RT range from emotional dysfunctions (e.g., anxiety, depression, bipolar disorders, irritability) to impairments in cognitive skills (e.g., learning, memory), from autism spectrum disorders to schizophrenia, but organic conditions like from atopic dermatitis to eye health were also considered. Oxidative stress, (neuro)inflammation, pyroptosis, apoptosis, disruption of the blood-brain barrier, epigenetic dysregulation, endoplasmic reticulum stress and altered neurotransmission systems are among the mechanisms underlying pollutants’ action on the brain discussed in the present Research Topic.

The Original Research articles published in this Research Topic represent a comprehensive set of experiments that not only establish correlations between environmental pollutants and neuropsychiatric disorders, but also propose an attempt to establish causal relationships and identify underpinning mechanisms. Contamination can be achieved by direct ingestion, or through air pollutants. Ingestion route is responsible of increased body levels of metals (Sun et al.; Mao et al.), pesticides (Men et al.) or microplastics (MPs) (Mohammadi et al.). In 2021, air pollution is second only to high blood pressure as a leading global risk factor for mortality (Du et al.; Zundel et al.). Clinical studies were performed on human cohorts from the Second People’s Hospital of Zhumadian (China) (Sun et al.), through local recruitment in the Detroit area (MI, USA) (Zundel et al.), or using the National Health and Nutrition Examination Survey (NHANES) database (Mao et al.; Men et al.; Du et al.). Several studies focused on escalating prevalence and burden of anxiety and depressive disorders since previous studies have indicated that environmental pollutants play a role in their onset and progression. In the present Research Topic, several Original Research articles confirm the strong correlation between pollutants and anxiety or depression and demonstrate lifelong effects, and marked sex-differences. Volatile compounds, such as ethylene oxide, increase the prevalence of depression particularly in males (Du et al.). Metals alone (Sun et al.) or in combination (Mao et al.) are significantly associated to both incidence and prevalence of depression, with gender differences highlighting higher prevalence in women. Barium and tin display the strongest correlation with the HAMD-24 scale that evaluates the severity of major depressive disorder (Sun et al.). The mixture of metals has been tested in the article by Mao et al., and the results indicate that cadmium is a key contributor to deleterious effects. Interestingly, the association between cadmium-containing metal mixtures, and exposure and prevalence of depression is mediated and potentialized by sleep disorders, involving both increased and reduced sleep duration.

Possible mechanisms underlying these effects of pollutants have drawn the attention of the authors who collectively underscore the role of inflammatory mechanisms. Small particulate matters (PM2.5) pass the Blood Brain Barrier and cause inflammation associated with mental health disorders, particularly anxiety and depression, in adolescents (Zundel et al.). The study of young people is a new field of particular relevance given the potential psychopathological consequences for the most vulnerable individuals. PM2.5 contamination is correlated with higher abundance of pro-inflammatory lipid mediators and IL6 cytokine. PM2.5 absorption. The inflammatory response differs between sexes, with PM2.5-related inflammation being higher in females, and results in a stronger association with anxiety disorders, even when the subjects were exposed at doses usually considered at lower risks. Effects of volatile compounds are mediated by blood cells, likely involve the immune system and result in inflammation (Du et al.). Polystyrene microplastics (PS-MPs) are capable of traversing the blood-brain barrier (BBB), accumulating within the brain, and inducing neurotoxicity through mechanisms that also involve inflammation (Mohammadi et al.). In turn, inflammation affects oxidative stress, and neurotransmitter activity (Mao et al.).

Pesticides, particularly organophosphates (OPP), facilitate the development of atopic dermatitis (AD), which is a chronic inflammatory skin condition, and their potential association is the focus of the study of Men et al. AD has increased significantly in developing regions in the last 30 years, particularly in children, thus strengthening the increased risk in the youth. OPP trigger oxidative stress and regulate inflammatory responses through the cholinergic pathway by interfering with cholinesterase activity, thereby affecting cytokine production and the expression of pro-inflammatory molecules. Dimethylphosphate (DMP) is the main contributor to AD and interestingly, it has also been involved in depression risk. Sex-differences may also exist since males showed higher DMP susceptibility than females, possibly due to sex differences in detoxifying enzyme activities.

One preclinical study on rat models investigates a possible strategy to mitigate pollutant-induced toxicity by regulating cellular processes. Mohammadi et al. assessed the role of MiR-103a-3p, a non-coding RNA regulating gene expression that is known to exert neuroprotection. The study demonstrates that miR-103a-3p reverses the cognitive deficits caused by exposure to PS-MPs in rats. This effect is mediated by reduction in inflammation, oxidative stress, apoptosis and pyroptosis. Moreover, miR-103-3p administration also inhibits signaling pathways responsible for endoplasmic reticulum stress that plays important roles in cognitive declines.

In a Community Case Study, Pedroza Carrillo et al. conducted a pilot study on brick kiln workers, which are known to be exposed not only to physical risk factors, but also to increased risk of health problems, including respiratory, musculoskeletal, gastrointestinal, reproductive, dermic, psychosocial disorders or complaints (1–3). This study revealed that living or working in brick kilns is associated with eye problems, including foreign body sensation, blurred vision and itchy eyes, watery eyes and eye discharge, photophobia, eye pain, decreased visual acuity and nyctalopia, as well as with a higher prevalence of self-reported symptoms, including irritability, anxiety, depression, and insomnia, highlighting the need for further mechanistic studies and long-term research on the ocular and neurological effects of brick kiln exposure.

The Original Research articles underline methodological limitations of the different studies, and propose future research axis. The analysis and their interpretation proved to be difficult since increased contamination may be correlated to higher or lower risk, depending on the pollutant in consideration. The predictive value also depends on the fluid that is scrutinized (e.g., serum vs urine) (Sun et al.). It will be necessary to identify the different inflammatory pathways activated by pollutants since they may lead to specific psychiatric outcomes (e.g., IL8 pathway correlates with risks of depression). The endocrine, cardiovascular, and nervous systems routes by which metals, plastics and other contaminants penetrate the organism need to be further explored since interactions between different physiological systems may strongly contribute to the severity of neurological and neuropsychiatric consequences.

Environmental exposures to several pollutants commonly present in different matrices (i.e., air, soil, water), including heavy metals, pesticides, herbicides, fertilizers, polycyclic or polynuclear aromatic hydrocarbons (PAH) and solvents, have been associated with adverse physical and mental health outcomes and premature deaths. Compelling epidemiological studies indicates that environmental contaminants brought into the air by the vaporization of volatile organic compounds and other anthropogenic pollutants might contribute, at least in part, to the development or progression of psychiatric disorders. Evidence comes predominantly from occupational work studies, with indoor activities being the most important sources of airborne pollutants affecting neural circuits implicated in mood disorders. To complete the Research Topic, 2 Reviews and 1 Mini Review analyzed the existing literature to provide important overviews on specific pollutant/mental disease associations.

Tota et al., for example, focused on the association of exposure to air (i.e., PM2.5, NO2, and SO2), soil and water pollutants (e.g., crude oil, heavy metals, agro-chemicals, polycyclic or polynuclear aromatic hydrocarbons, solvents) with an increased risk for anxiety, depression, schizophrenia, and autism spectrum disorders, and provided an overview of the potential underlying patho-mechanisms involved, including increased oxidative stress, systematic inflammation, disruption of the blood-brain barrier, and epigenetic dysregulation. Importantly, authors included light and noise pollution that are underestimated concerns for citizens in urbanized areas, but actually are correlated with an increased risk of neurodegenerative disorders, particularly Alzheimer’s disease. Authors conclude by reviewing evidence associating extreme weather conditions, such as hurricanes, floods, wildfires, and droughts, with long-term adverse mental conditions, especially with symptoms of depression, anxiety and post-traumatic stress disorder (PTSD).

In their Review, Gallardo et al. examined the current literature to find an answer to a very simple question: can air quality and residential settings (i.e., urban vs rural) be key factors contributing to cognitive decline and mood disorders associated with aging? From the evidence collected so far, authors concluded that air pollutants not only affect cognitive function and increase the risk of dementia and neurodegenerative disorders, but they also appear to be associated with a higher risk for depression, anxiety and suicide. Older adults seem particularly susceptible and vulnerable to the effects of particulate matter pollution, but relationships, social inclusion and activities can modulate and mitigate the effects observed during the aging in subjects living in rural areas that are exposed to less contamination, in line with the finding that limited or poor social connections can elevate the risk of dementia (4).

To sort out how indoor exposure to airborne pollutants affect brain functionality, Torres et al. proposed a systematic PubMed literature Mini Review on the potential association of indoor pollutants with structural and functional changes of neural networks specialized in emotional and cognitive behavioral underpinnings. Specifically, they examined first the nature of indoor airborne pollutants and toxicity mechanisms, including oxidative stress, inflammation, and activation of neural circuits. Then, they reviewed the impact of indoor airborne pollutants on the epidemiology of mood disorders and discussed appropriate measures for reducing the adverse effects of indoor airborne pollutants on brain-signaling pathways.

As for the 6 Original Research articles, clear gender-dependent effects of pollutants on mental diseases are widely reported in the literature. Gallardo et al. observed that some behavioral effects induced by air pollution depend on the gender of the subjects, with women appearing more vulnerable than men, in line with studies reviewed by Tota et al. showing that females are more susceptible to air pollution than men (5) and show a stronger association of air pollution with increased risk of hospital admissions for schizophrenia (6), although no gender-differences were observed in the positive association between SO2 and NO2 and increased outpatient anxiety visits in a hospital-based study in northwestern China (7). Notably, females are at higher risk of mental diseases also when exposed to extreme weather events like floods, hurricanes, wildfires, and droughts (8). Consistently, Torres et al. reported evidence that household air pollutants affect women’s health more broadly than men’s, particularly in low-income settings (9).

In line with the March 2026 report from the European Environment Agency (EEA, https://www.eea.europa.eu/en/analysis/publications/pollution-and-mental-health-current-scientific-evidence), articles collected in this Research Topic confirm that long-term exposure to air pollution and to other chemical contaminants are significantly linked to increased risks of mental diseases, underscoring the urgent need for judicial authorities to enforce existing environmental regulations and pollution policies to safeguard workers and residents in high-risk areas.
